# 

**Published:** 1844-06

**Authors:** 


### CONTENTS.

Page

### LIBRARY.

A Treatise on the Anatomy and Physiology of the Teeth, &c. By David Wemyss Jobson, M. R. C. S. E., Dentist in ordinary to His Majesty, and to His Royal Highness the Duke of Sussex, &c.,.................................................. 73

### JOURNAL.

ARTICLE I.—Obituary Notice of Prof. Horace H. Hayden, being a Valedictory Address to the Graduating Class of the Baltimore College of Dental Surgery. By Prof. T. E. Bond, Jr., M. D., 221

ARTICLE II.—An Essay on the Morbid Effects of Diseased Teeth upon the Human System. By W. W. H. Thackston, D. D. S. 230

ARTICLE III.—Hare-lip, and its Treatment. By Dr. S. P. Hullihen, Wheeling, Va.......................................... 244

ARTICLE IV.—Compound Root Forceps. By Dr. S. P. Hullihen, Wheeling, Va.......................................... 254

ARTICLE V.—Mineral Paste for Filling Teeth,.................... 256

ARTICLE VI.—Address delivered before the Cincinnati Association of Dental Surgeons. By M. Rogers, M. D.,................ 258

ARTICLE VII.—Letter from C. L. Norton, Dentist of New York, to the Baltimore Editor,.......................................... 266

ARTICLE VIII.—Tartar, or Calculus of the Mouth. Translated from the French of Delabarre. By * A *....................... 268

ARTICLE IX.—The Apparel destined by Nature for the Absorption of the Roots of the Temporary Teeth. Translated from the French of Delabarre. By * A *........................... 279

ARTICLE X.—Quackery.............................................. 289

### COLLECTANEA.

The Burning Wells of Kenawha,...................................... 294

Cyst in the orbital cavity,.......................................... 296

Filing the Teeth,.................................................... 297

Rupture of the Trachea,.............................................. 298

### MISCELLANEOUS NOTICES.

Baltimore College of Dental Surgery,................................ 298

To Correspondents,................................................... 300

To our Subscribers,.................................................. 300

Gossip with our Profession, by the Junior Editor,................... 300

